# Palliative sedation in Germany: factors and treatment practices associated with different sedation rate estimates in palliative and hospice care services

**DOI:** 10.1186/s12904-018-0303-7

**Published:** 2018-03-13

**Authors:** Stephanie Stiel, Mareike Nurnus, Christoph Ostgathe, Carsten Klein

**Affiliations:** 1 Department of Palliative Medicine, University Hospital Erlangen, Friedrich-Alexander-Universität Erlangen-Nürnberg, Erlangen, Germany; 2 Institute for General Practice, Hannover Medical School, Carl-Neuberg-Straße 1, 30625 Hannover, Germany

**Keywords:** Palliative sedation, Framework, Standards, Drug monitoring, Symptom management, physician’s practice patterns

## Abstract

**Background:**

Clinical practice of Palliative Sedation (PS) varies between institutions worldwide and sometimes includes problematic practices. Little available research points at different definitions and frameworks which may contribute to uncertainty of healthcare professionals in the application of PS. This analysis investigates what demographic factors and characteristics of treatment practices differ between institutions with high versus low sedation rates estimates in Palliative and Hospice Care in Germany.

**Methods:**

Data sets from 221 organisations from a prior online survey were separated into two sub-groups divided by their estimated sedation rate A) lower/equal to 16% (*n* = 187; 90.8%) and B) higher than 16% (*n* = 19; 9.2%) for secondary analysis. Demographic factors and characteristics of PS treatment practices between the two groups were compared using T-Tests and Chi^2^/ Fisher Exact Tests and considered significant (*) at two-sided *p* < .05.

**Results:**

Organisations in group B report that they discuss PS for a higher proportion of patients (38.5%/10.2%, *p* < 0.000**), rate agitation more often as an indications for PS (78.9%/ 53.5%, *p* = 0.050*), and are more likely to use Lorazepam (63.2%/ 37.4%, *p* = 0.047*), Promethazin (26.3%/ 9.6%, *p* = 0.044*), and (Es-)Ketamin (31.6%/ 12.8%, *p* = 0.039*) than representatives in group A. Both groups differ significantly in their allocation of three case scenarios to different types of PS.

**Conclusions:**

Both definitions and patterns of clinical practice between palliative and hospice care representatives show divergence, which may be influenced one by another. A comprehensive framework considering conceptual, clinical, ethical, and legal aspects of different definitions of PS could help to better distinguish between different types and nuances of PS.

## Background

Despite holistic Palliative Care (PC) some patients experience unbearable suffering. Controlled ‘Palliative sedation’ (PS) can be an option for these patients [1]. PS has been defined as “the monitored use of medications intended to induce a state of decreased or absent awareness (unconsciousness) in order to relieve the burden of otherwise intractable suffering […]” [2]. Hence uniformity on the understanding of PS is suggested by the definition, PS refers to a number of different practices which can vary in terms of intensity, time, and intended effects.

The (inter-)national evidence on indications [3], medications and dosages [4–6], and duration of PS in specialist palliative care (SPC) [7] is multifaceted. Studies concentrate on team attitudes [8], emotional burden [9] and experiences especially with continuous deep PS until the patient’s death [10]. Several (inter-)national guidelines/frameworks on PS have been published [11–13] including one from the European Association for Palliative Care (EAPC) [2]. However, the clinical practice still varies between institutions worldwide and so far no general clinical standard has been established.

A German questionnaire survey by the authors’ working group confirmed numerous and substantial differences in the reported PS practices between different palliative and hospice care institutions [14, 15]. In this study, “*the estimated frequency of PS ranges from 0 to 80% of all patients treated per year (mean 6.7%) and some PC specialists report discussing PS as treatment option for every patient they encounter*” [14]. The causes for these differences are not well explained yet.

Some of the differences may be due to problematic use of the measure. There is evidence that in single cases PS may be used (i) inappropriately and too hasty while neglecting multidimensional assessment and possible therapeutic options for patients [16], applying inadequate medications such as opioids for PS [17, 18], to alleviate staff members’ burden [19] in decision-making for PS (ii) under delicate and sensitive indications such as requests for euthanasia [20], hence other therapeutic options than PS would be applicable [21], or due to family caregivers’ wish for PS [16], or even (iii) abusively by physicians with the primary goal of hastening the patient’s death [22, 23]. All these problematic practices should be taken seriously to prevent mainly patients but also family caregivers and healthcare professionals from harm.

The available data addressing these clinical variations in PS already reveal a major challenge. The reported variation of sedation practices may be based at least partially on different understandings and definitions of PS which might be closely connected to a lack of widely accepted terminology and a consistent framework [24, 25]. These theoretical variations may result in uncertainty in the practical application of PS for healthcare professionals and might foster problematic practices.

To date there is a lack of comprehensive data and specific analyses that explain the substantially deviant sedation rates and differing treatment practices of PS in Germany.

### Study aim

This secondary data analysis of a previous study from 2012, aimed to find and describe differences in clinical factors, ratings of case scenarios, and characteristics in institutions with high versus low sedation rates estimates in Palliative and Hospice Care in Germany. The findings will add to the discussion of different PS practices.

## Methods

### Study material & participants

In 2012, an internally developed online questionnaire consisting of 34 questions divided into seven content sections (estimated number of patients, indications, monitoring, documentation, clinical practice, guidelines, and demographics) was sent out to 605 heads of palliative care units (PCU), hospices (H), specialized palliative home care services for adults (SPHC), and specialized palliative home care services for children (SPPHC) listed in the German official address registers. They were asked to give information about their organisations’ usual clinical practice of PS reflecting a period of 1 year [14].

The initial survey included seven written case scenarios displaying different patterns of PS which were rated by the organisations as PS (“yes”), no PS (“no”) or undecided (“unsure”) (for original scenario description see Table 2). The case scenario descriptions were constructed in order to cover the different forms of PS [26].These cases were intended to better understand the individual definition of PS the clinicians work with.

A) Symptom oriented treatment resulting in sedation, may also be PS to alleviate otherwise intractable suffering (symptom: agitation)
B) Symptom oriented treatment resulting in sedation (symptom: delirium)
C) Symptom oriented treatment resulting in sedation (symptom: pain due to muscle cramps)
D) secondary sedative effect of other medication not primarily intended for PS (symptom: pain)
E) PS to alleviate otherwise intractable suffering (symptom: nausea)
F) PS in the dying phase due to an acute event (symptom: bleeding)
G) intermittent PS for symptom relief (symptom: existential suffering)

Overall, 225 questionnaires were returned resulting in a response rate of 37.2% of all invited representatives. Four data sets had to be excluded due to inconsistencies, so that *n* = 221 data sets were considered for further analysis. The overall frequency of the use of PS reported by these 221 participating organisations varied from 0 to 80% (mean 6.7%; SD ±9.3%; median 3.8%; *n* = 206; *n* = 15 missing values [14].

### Statistical analysis

IBM SPSS Statistics 21 (SPSS Inc., Chicago, IL, USA) for Windows was used for statistical analysis.

For the analysis at hand, first, all data sets were allocated to two sub-groups divided by their estimated low versus high sedation rate. This sub-group classification is based on statistical suggestions regarding the actual empirical data distribution from the former survey study. The cut-off point was set to the sum of the mean value (6.7%) plus one SD (9.3%) to contrast the most striking outlier data of organisations’ estimated PS prevalence effectively [14]. The two established groups are A) estimated sedation rate is lower /equal to 16% (*n* = 187 organisations; 90.8%) and B) estimated sedation rate is higher than 16% (*n* = 19 organisations; 9.2%). Descriptive data (mean, median, standard deviation (SD), range from minimum to maximum) for the description of the characteristics of the total sample and the two sub-groups as well as treatment practices were calculated.

Second, to compare demographic factors, characteristics of treatment practices, and ratings of seven case scenarios (see Table 2) between these two groups T-Tests were performed in variables with interval or ratio scales, Chi^2^/ Fisher Exact Tests in variables with nominal or ordinal scales. All tests were considered significant (*) at two-sided *p* < .05.

### Ethics

The study plan was presented to the ethics committee of the medical faculty of Erlangen. A formal approval was not regarded to be necessary.

## Results

### What are the differences in treatment practices between participating organisations with high versus low sedation rate estimates?

Organisations in group B (high estimated sedation rate) reported that

i) they discussed PS as an option for therapy in a significantly higher proportion of patients (38.5% versus 10.2%, *p* < 0.000**),
ii) the rate of agitation as one of the four most frequent indications for PS was significantly higher (78.9% versus 53.5%, *p* = 0.050*),
iii) they evaluate symptoms during PS significantly less often (never: 5.6% versus 0%; sometimes: 0% versus 2.8%; mostly: 11.1% versus 8.5%; always: 83.3% versus 88.7%, *p* < 0.015*),
iv) they were significantly more likely to use Lorazepam (=Tavor®) (63.2% versus 37.4%, *p* = 0.047*), Promethazin (=Atosil®) (26.3% versus 9.6%, *p* = 0.044*), and (Es-)Ketamin (=Ketanest®) (31.6% versus 12.8%, *p* = 0.039*),
v) they planned significantly different time intervals until weaning phases during PS (< 12 h: 41.7% versus 13.3%; 12–24 h: 25.0% versus 61.1%; > 24 h: 33.3% versus 25.7%, *p* = 0.017*)

than representatives of organisations in group A (low estimated sedation rate) (see Table 1).

**Table 1:** Differences of ratings between Group A and Group B to online survey on Palliative Sedation calculated by ^a^T-Test; ^b^Fisher Exact Test; ^c^Chi^2^ test (all considered significant at *p* < 0.05*)

Questionnaire section	Item	Group A: low sedation rate estimates (n)		Group B: high sedation rate estimates (n)	*p*
1 Prevalence	^a^PS discussed as an option for therapy	10.20% ± 9.41% (183)		38.49% ± 22.08% (19)	0.000**
	^a^Patients competent to consent to PS	51.65% ± 37.78% (183)		52.58% ± 28.57% (19)	0.917
2 Indications	^b^Agitation	53.5%		78.9%	0.050*
	^b^Dyspnea, ^b^Epileptic seizure, ^b^Physical exhaustion, ^b^Acute bleeding, ^b^Anxiety, ^b^Pain, ^b^Depressiveness, ^b^Delirium, ^b^Nausea/Vomiting, ^b^Existential suffering				> 0.143
3 Evaluation	^c^ Evaluation of level of consciousness during PS (*n* = 179/18)	Never	2.2%	5.6%	> 0.570
		Sometimes	5.0%	11.1%	
		Mostly	21.2%	16.7%	
		Always	71.6%	66.6%	
	^c^ Evaluation of symptoms during PS (*n* = 177/18)	Never	0.0%	5.6%	0.015*
		Sometimes	2.8%	0.0%	
		Mostly	8.5%	11.1%	
		Always	88.7%	83.3%	
	^b^Way of evaluating depth of sedation (monitoring, response, touching, pain stimulus, appraisal of vital signs, dose rate of drugs, response of close ones)				> 0.079
	^c^How often level of consciousness/symptoms are evaluated				0.509
	^c^Who conducts evaluation				0.882
	^b^Use of scores for evaluation of level of consciousness (Richmond-Agitation-Sedation-Score, Ramsay-Sedation-Score, Agitation Distress Scale)				> 0.208
	^b^Use of scores for evaluation of level of symptoms (VAS/NRS/VRS, Edmonton Symptom Assessment System, Minimal Documentation System, symptom and problem checklist from HOPE)				> 0.228
4 Documentation	Areas of documentation before sedation				> 0.122
	^b^Indication for conduction of PS; ^b^previous attempts of treatment; ^b^process of decision-making; ^b^aspired depths of sedation; ^b^aspired length of sedation				
	Area of documentation during sedation:				> 0.233
	^b^drugs, dosage and application method; ^b^vital signs; ^b^transmitting of alimentation/liquids; ^b^other drugs/medical measures				
5 Treatment Strategies	Drugs used for PS (187/19):				
	^b^Lorazepam (=Tavor®)		37.4%	63.2%	0.047*
	^b^Promethazin (=Atosil®)		9.6%	26.3%	0.044*
	^b^(Es-)Ketamin (=Ketanest®)		12.8%	31.6%	0.039*
	^b^Haloperidol (=Haldol®), ^b^Clonazepam (=Rivotril®), ^b^Flunitrazepam (=Rohypnol®), ^b^Midazolam (=Dormicum®), ^b^Propofol (=Disoprivan®), ^b^Levomepromazin (=Neurocil®), ^b^Opiates, ^b^Melperon (=Eunerpan®), ^b^muscle relaxants				> 0.128
	Way of regulating of level of consciousness				> 0.554
	^c^Aiming for weaning phases after what length of sedation (133/12):				
	< 12 h		13.3%	41.7%	0.017*
	12–24 h		61.0%	25.0%	
	> 24 h		25.7%	33.3%	
	Artificial hydration/nutrition:				> 0.763
	^c^Patients competent to consent to therapy decide on artificial hydration/nutrition; ^c^Independent from decision PS; ^c^ Belongs to basic supply; ^c^withhold				
6 Guidelines	^b^Internal guidelines/instructions for PS available				> 0.855
	^b^Knowledge of (inter-)national guidelines				> 0.624
	^c^Extent of consideration of guidelines				> 0.539

* and bold numbers are with significance

### What are the differences in the ratings of case scenarios (see Table 2) between participating organisations with high versus low sedation rate estimates?

Organisations in group B (high sedation rate)

i) rated scenario D (representing secondary sedative effect of other medication not primarily intended for PS) significantly more often as PS (29.4% versus 8.5%, *p* = 0.025*),
ii) rated scenario F (representing PS in the dying phase due to an acute event) significantly more often as PS (100% versus 64.2%, *p* = 0.011*),
iii) rated scenario G (representing intermittent PS for symptom relief) significantly less often as PS (70.6% versus 79.6%, *p* = 0.042*)

**Table 2:** Differences in the distribution of percentages (%) of answers of Group A and Group B to case scenarios (Chi^2^ Test; *p* < 0.05 considered significant^*^)

7) Definition: Case scenario	Rating	Group A	Group B	*p*
[A] To relief a patients agitation through clouded awareness he receives over 24 h 48 mg Midazolam (=Dormicum®) through a syringe driver. During this treatment, which is continued right up to his death, it is impossible to carry on a conversation with him.	no	14.3%	17.7%	.536
	yes	66.1%	52.9%	
	unsure	19.6%	29.4%	
		(*n* = 168)	(*n* = 17)	
[B] A delirious patient is treated with 2 mg Haloperidol (=Haldol®) for every 6 h. Under this dosage, which is necessary for a successful treatment, he seems obviously sedated.	no	84.4%	76.5%	.660
	yes	6.6%	11.8%	
	unsure	9.0%	11.7%	
		(*n* = 167)	(*n* = 17)	
[C] Under the medical indication of pronounced muscle cramps a patient receives circadian 10 mg Diazepam (=Valium®) daily. Symptoms are soothed through the consequential sedation. Medication is continued for several days and nights. Despite somnolence, patient can welcome and greet visitors, until he finally passes away.	no	73.6%	64.7%	.703
	yes	16.2%	23.5%	
	unsure	10.2%	11.8%	
		(*n* = 167)	(*n* = 17)	
[D] A patient suffering from intense pain needs a daily dose of 300 mg Morphium s.c.. Under this medication he appears exceedingly somnolent and sedated, but can be awakened through loud address.	no	80.0%	64.7%	**.025** ^*^
	yes	8.5%	29.4%	
	unsure	11.5%	5.9%	
		(*n* = 165)	(*n* = 17)	
[E] Because of not otherwise manageable nausea the patient is treated with 2.5 mg Lorazepam (=Tavor®) every 8 h. Under this therapy patient is sleeping. During two attempts of reducing the dose he declares that the nausea persists. Therefore medication is increased again. Few hours after adjustment of dose he passes away.	no	36.6%	29.4%	.733
	yes	48.8%	58.8%	
	unsure	14.6%	11.8%	
		(*n* = 164)	(*n* = 17)	
[F] Due to dinstinctive and severe life-threatening tumour haemorrhage and the fulminant panic attack a patient receive 10 mg Midazolam (=Dormicum®) i.v.	no	23.5%	0.0%	**.011** ^*^
	yes	64.2%	100.0%	
	unsure	12.3%	0.0%	
		(*n* = 162)	(*n* = 17)	
[G] According to the wish of a patient she receives under indication of existential suffering 3 mg/h Midazolam (=Dormicum®). To determine patients wish again, dose is reduced after 24 h. As patient does not want a continuation of medication it is weaned.	no	10.5%	29.4%	**.042** ^*^
	yes	79.6%	70.6%	
	unsure	9.9%	0.0%	
		(*n* = 162)	(*n* = 17)	

* and bold numbers are with significance

than representatives of organisations in group A (low sedation rate) (see Table 2).

### Differences in demographic staff data between organisations with low versus high sedation rate estimates

The percentage of physicians specialised in internal medicine was by trend higher in group A with a low sedation rate estimate (32.4% versus 10.5%, *p* = 0.64). The percentage of physicians specialised in anaesthesiology, neurology, general medicine, and radiotherapy was equally distributed in both groups (*p* > 0.237).

Neither group differed in terms of sex, age, professional group, years of professional experience and years of professional experience in palliative and hospice care (*p* > 0.085). No significant differences were found in the distribution of palliative and hospice care settings within both groups, the proportion of representatives from inpatient hospices, paediatric and adult community palliative care teams, and the proportion of inpatient and outpatient care setting representatives (*p* > 0.338).

## Discussion

The study at hand aimed at identifying possible factors of palliative and hospice care representatives and characteristics of treatment practices associated with differences between certain institutions that estimated a high versus low sedation rate in their service in a survey on PS in Germany.

The starting point and findings reported here show that there were considerable differences between palliative and hospice care services first in their sedation rate estimates, second in their clinical PS practices and third in their definitions of different PS practices.

The estimated prevalence of PS found in our questionnaire study [14] has been confirmed by data of other studies in Germany. Three papers report prevalence rates between 7 and 34% [3, 27, 28]. Compared to German findings, an international review by Maltoni et al., however, found an even higher range between sedation rates from 14.6% to 66.7% among 1.807 consecutive patients from 10 different studies [29]. Different clinical practices of PS in palliative and hospice care in Germany resulting from the presented analysis affect in particular indications for PS, frequency of symptom evaluation during PS, the usage of drugs, and planned weaning phases during PS in this analysis. These deviations are also reflected in and in accordance with the current international research literature which reports a wide range of indications for PS [4, 29], different medications and dosages [5, 6], and patterns of evaluation and monitoring PS [30]. The divergence in clinical practice seems to be associated with different understandings of PS [31–33]. Several authors already pointed at a lack of accepted terminology and a consistent framework which makes it difficult to describe different PS practices [25, 34]. Even different frameworks that should assist as guidance vary considerably in content and quality [35]. The parent study to this analysis [14] showed that a considerable percentage of institutional representatives were not aware of the EAPC framework [2] or its German translation [36].

Our analysis supports the notion that palliative and hospice care professionals do not agree on a clear definition of PS versus a loose collective term for numerous nuances and types of PS. The case scenarios were assigned to varying definitions of PS and the assignment seem to have an impact on the estimated sedation rat in the service. This raises the questions whether due to these multi-layered and broad variances, palliative and hospice care practitioners may experience uncertainty in clinical PS practice. In consequence, the preparation, discussion, and decision making prior to PS, the evaluation and documentation of PS and the assessment of PS outcomes are set into practice very differently [14, 15]. In particular misunderstandings on definition of PS may foster problematic practices, which should be prevented.

This study shows no clear signs of associations between health care professionals’ demographic data and profession-related background on the one hand and their PS practice of high versus low sedation rate on the other.

### Study limitations

The results of the study are somewhat limited by a rather low response rate to the questionnaire of the main study, a lack of information about possible non-response bias and by an unequal percentage of representatives from all palliative and hospice care settings. These limitations may constrain the generalizability of the data.

Furthermore, answers to the questionnaire may include estimation errors. This uncertainty about the extent to which the views of the interviewees are representative of the population of German palliative medicine specialists is compounded by the uncertainty about the extent to which the responses reflect actual care delivery.

The rationale for determining the cut point distinguishing low vs. high estimated PS rates is based on statistical suggestions regarding the data distribution from the former survey study. The cut-off point was set to the sum of the mean value (6.7%) plus one SD (9.3%) to contrast effectively the striking outlier data. To our knowledge no workgroup or publication has yet defined a reasonable cut point for low vs. high estimated PS rates and data on PS prevalence differ considerably, so that this is a whole new approach to extend evidence. This rationale and missing comparable approaches challenge the interpretation of the group comparisons, as does the inability to perform multivariable analysis. Nonetheless, differences in treatment practice are tangible, but cannot allow statistical inference for the entire population of German PC institutions.

From a methodological point of view, the number of data sets in each group (A versus B) was distributed unequally. A certain bias may derive from the allocation of the low and high sedation rate groups, because the cut-off was set considering clinical, empirical and scientific arguments.

## Conclusions

The analysis presented here gives new impetus to describing clinical differences in PS practice and imbedding them in a broader context. Both definitions and patterns of clinical practice between palliative and hospice care representatives show divergence, which may be closely interrelated and mutually dependent.

More research is needed (i) to describe existing differences in definitions and clinical patterns of PS between care practitioners, in order to (ii) better explain the investigated divergence and the relation between certain aspects of PS. A comprehensive practical framework considering conceptual, clinical, ethical, and legal aspects of different definitions of PS could help to better distinguish between different types and nuances of PS.

This may help in long-term to minimise problematic practice and prevent patients, family caregivers and healthcare professionals from harm.

## Abbreviations

EAPC: European Association for Palliative Care
H: Hospices
PC: Palliative care
PCU: Palliative care units
PS: Palliative sedation
SD: Standard deviation
SPHC: Specialized palliative home care services for adults
SPPHC: Specialized palliative home care services for children
